# Supplementary material for: A randomized, controlled Phase 1b trial of the Sm-TSP-2 Vaccine for intestinal schistosomiasis in healthy Brazilian adults living in an endemic area
Source: PLoS Negl Trop Dis. 2023 Mar 30;17(3):e0011236. doi: 10.1371/journal.pntd.0011236 (PMC10089325; doi:10.1371/journal.pntd.0011236)
Supplement: S1 Consort checklist — (PDF) [file pntd.0011236.s001.pdf]

CONSORT 2010 checklist of information to include when reporting a randomised trial*

Section/Topic	Item No	Checklist item	Reported on page No
**Title and abstract**			
	1a	Identification as a randomised trial in the title	1
	1b	Structured summary of trial design, methods, results, and conclusions (for specific guidance see CONSORT for abstracts)	1,3,4
**Introduction**			
Background and objectives	2a	Scientific background and explanation of rationale	6-7
	2b	Specific objectives or hypotheses	8
**Methods**			
Trial design	3a	Description of trial design (such as parallel, factorial) including allocation ratio	9
	3b	Important changes to methods after trial commencement (such as eligibility criteria), with reasons	n/a
Participants	4a	Eligibility criteria for participants	9-10
	4b	Settings and locations where the data were collected	9-10
Interventions	5	The interventions for each group with sufficient details to allow replication, including how and when they were actually administered	8-10
Outcomes	6a	Completely defined pre-specified primary and secondary outcome measures, including how and when they were assessed	11-15
	6b	Any changes to trial outcomes after the trial commenced, with reasons	n/a
Sample size	7a	How sample size was determined	n/a (Phase 1)
	7b	When applicable, explanation of any interim analyses and stopping guidelines	11
Randomisation:			
Sequence generation	8a	Method used to generate the random allocation sequence	11
	8b	Type of randomisation; details of any restriction (such as blocking and block size)	11
Allocation concealment mechanism	9	Mechanism used to implement the random allocation sequence (such as sequentially numbered containers), describing any steps taken to conceal the sequence until interventions were assigned	11
Implementation	10	Who generated the random allocation sequence, who enrolled participants, and who assigned participants to interventions	11
Blinding	11a	If done, who was blinded after assignment to interventions (for example, participants, care providers, those	11

		assessing outcomes) and how	
	11b	If relevant, description of the similarity of interventions	n/a
Statistical methods	12a	Statistical methods used to compare groups for primary and secondary outcomes	15-17
	12b	Methods for additional analyses, such as subgroup analyses and adjusted analyses	n/a
**Results**			
Participant flow (a diagram is strongly recommended)	13a	For each group, the numbers of participants who were randomly assigned, received intended treatment, and were analysed for the primary outcome	Fig. 1
	13b	For each group, losses and exclusions after randomisation, together with reasons	18
Recruitment	14a	Dates defining the periods of recruitment and follow-up	18
	14b	Why the trial ended or was stopped	n/a
Baseline data	15	A table showing baseline demographic and clinical characteristics for each group	Table 1
Numbers analysed	16	For each group, number of participants (denominator) included in each analysis and whether the analysis was by original assigned groups	Table 2, Figs 2-3
Outcomes and estimation	17a	For each primary and secondary outcome, results for each group, and the estimated effect size and its precision (such as 95% confidence interval)	18-31
	17b	For binary outcomes, presentation of both absolute and relative effect sizes is recommended	Figs 2-3
Ancillary analyses	18	Results of any other analyses performed, including subgroup analyses and adjusted analyses, distinguishing pre-specified from exploratory	n/a
Harms	19	All important harms or unintended effects in each group (for specific guidance see CONSORT for harms)	18-24
**Discussion**			
Limitations	20	Trial limitations, addressing sources of potential bias, imprecision, and, if relevant, multiplicity of analyses	33-34
Generalisability	21	Generalisability (external validity, applicability) of the trial findings	35
Interpretation	22	Interpretation consistent with results, balancing benefits and harms, and considering other relevant evidence	31-35
**Other information**			
Registration	23	Registration number and name of trial registry	9
Protocol	24	Where the full trial protocol can be accessed, if available	n/a
Funding	25	Sources of funding and other support (such as supply of drugs), role of funders	Included in online submission
