# Supplementary figures and images for: A randomized, controlled Phase 1b trial of the Sm-TSP-2 Vaccine for intestinal schistosomiasis in healthy Brazilian adults living in an endemic area
Source: PLoS Negl Trop Dis. 2023 Mar 30;17(3):e0011236. doi: 10.1371/journal.pntd.0011236 (PMC10089325; doi:10.1371/journal.pntd.0011236)

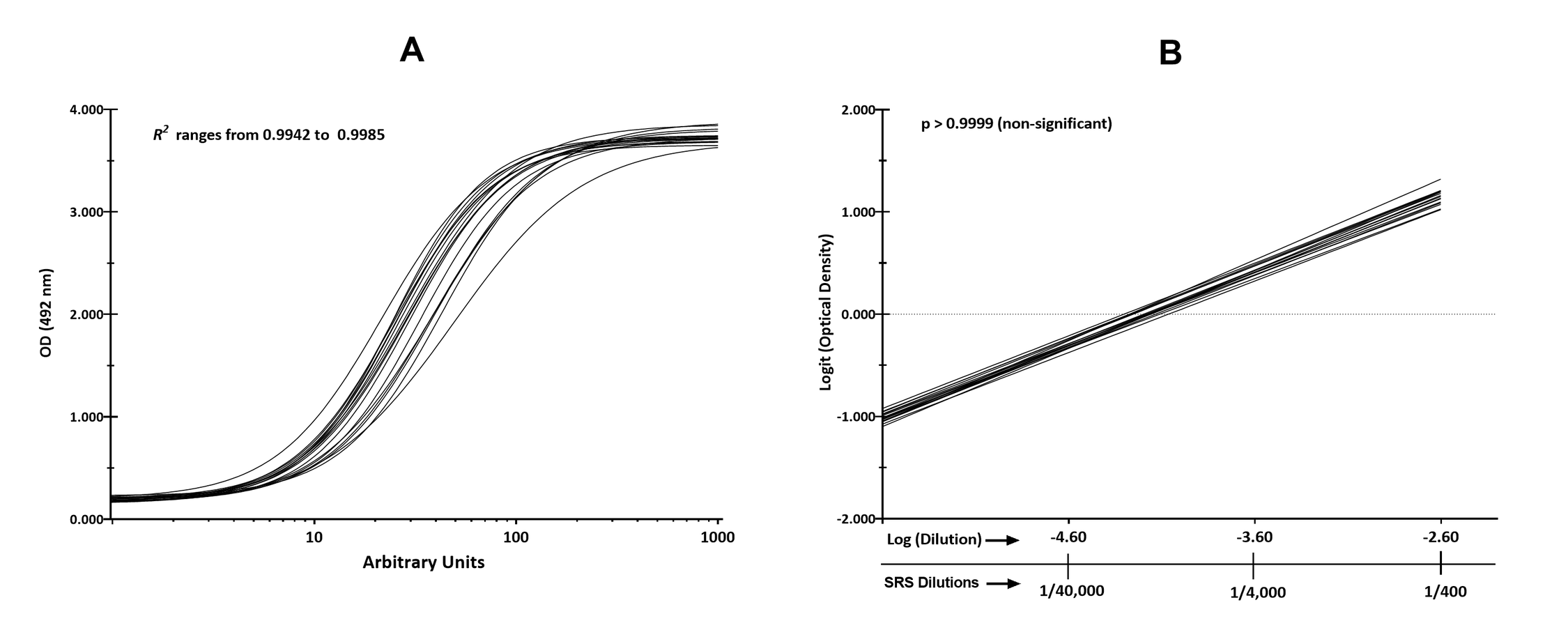

Supplement: S1 Fig — ELISA quality control: (A) 16 standard calibration curves (SCCs) generated from each ELISA plate were plotted along a four-parameter logistic log scale, where the X-axis represents the log of the dilution of SRS in Arbitrary Units (AU) and the Y-axis its Optical Density (OD) at 492nm. SRS = Standard Reference Serum; (B) Parallelism test: linearization of the 16 SCCs shown in panel (A) using a logit-log scale. The X-axis represents the log of the dilution and the Y-axis represents the fully specified logit of OD492nm. Tests of parallelism were performed using an ANOVA test, which indicated no significant departure from parallelism (p>0.9999). (TIF) [file pntd.0011236.s002.tif]

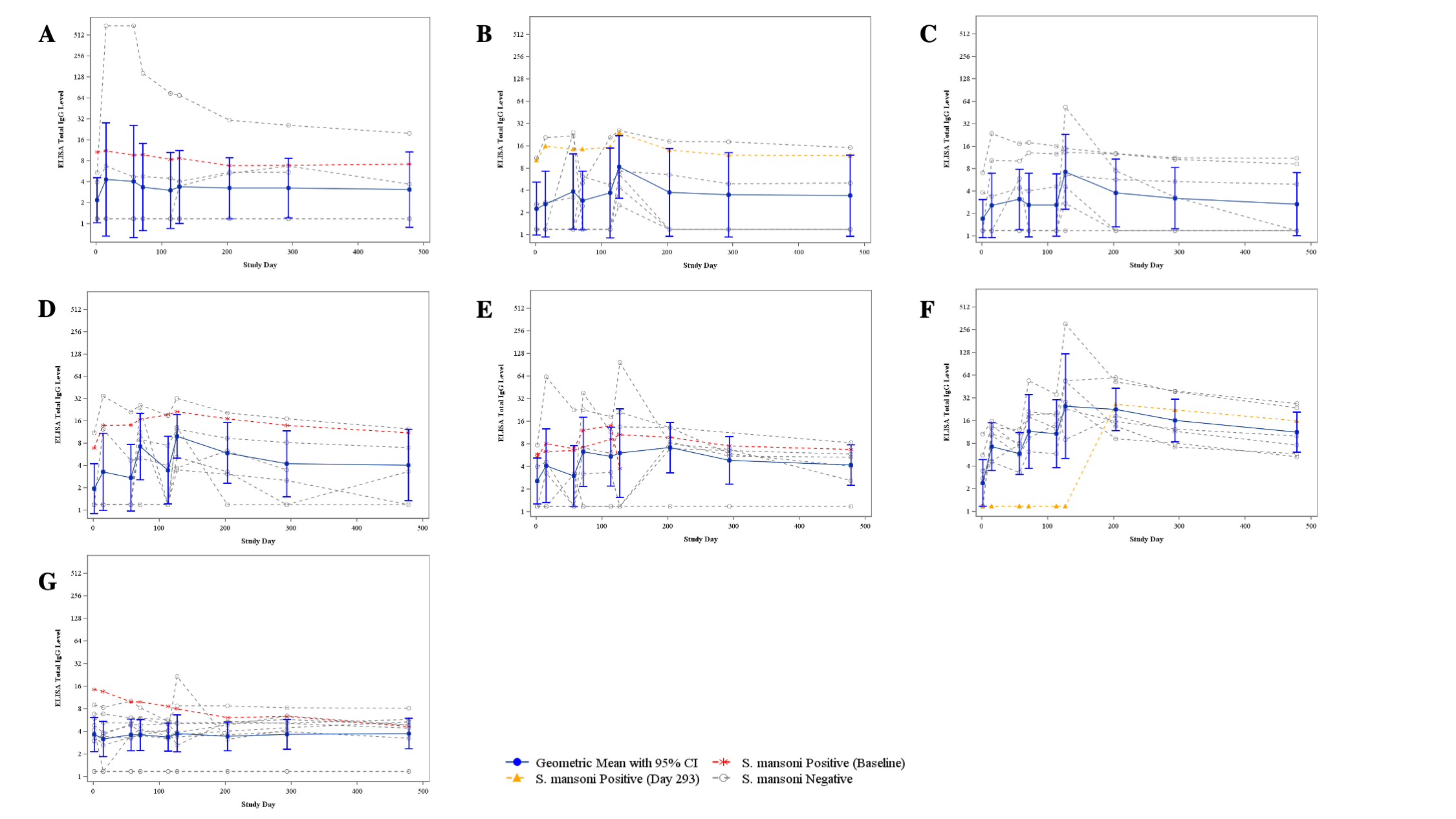

Supplement: S2 Fig — Individual study participant anti-Sm-TSP-2 ELISA IgG values over time, by S. mansoni infection status at baseline and at study day 293: (A) 10 μg Sm-TSP-2/Alhydrogel; (B) 10 μg Sm-TSP-2/Alhydrogel with AP 10–701; (C) 30 μg Sm-TSP-2/Alhydrogel; (D) 30 μg Sm-TSP-2/Alhydrogel with AP 10–701; (E) 100 μg Sm-TSP-2/Alhydrogel; (F) 100 μg Sm-TSP-2/Alhydrogel with AP 10–701; (G) Euvax Hepatitis B vaccine. (TIF) [file pntd.0011236.s003.tif]

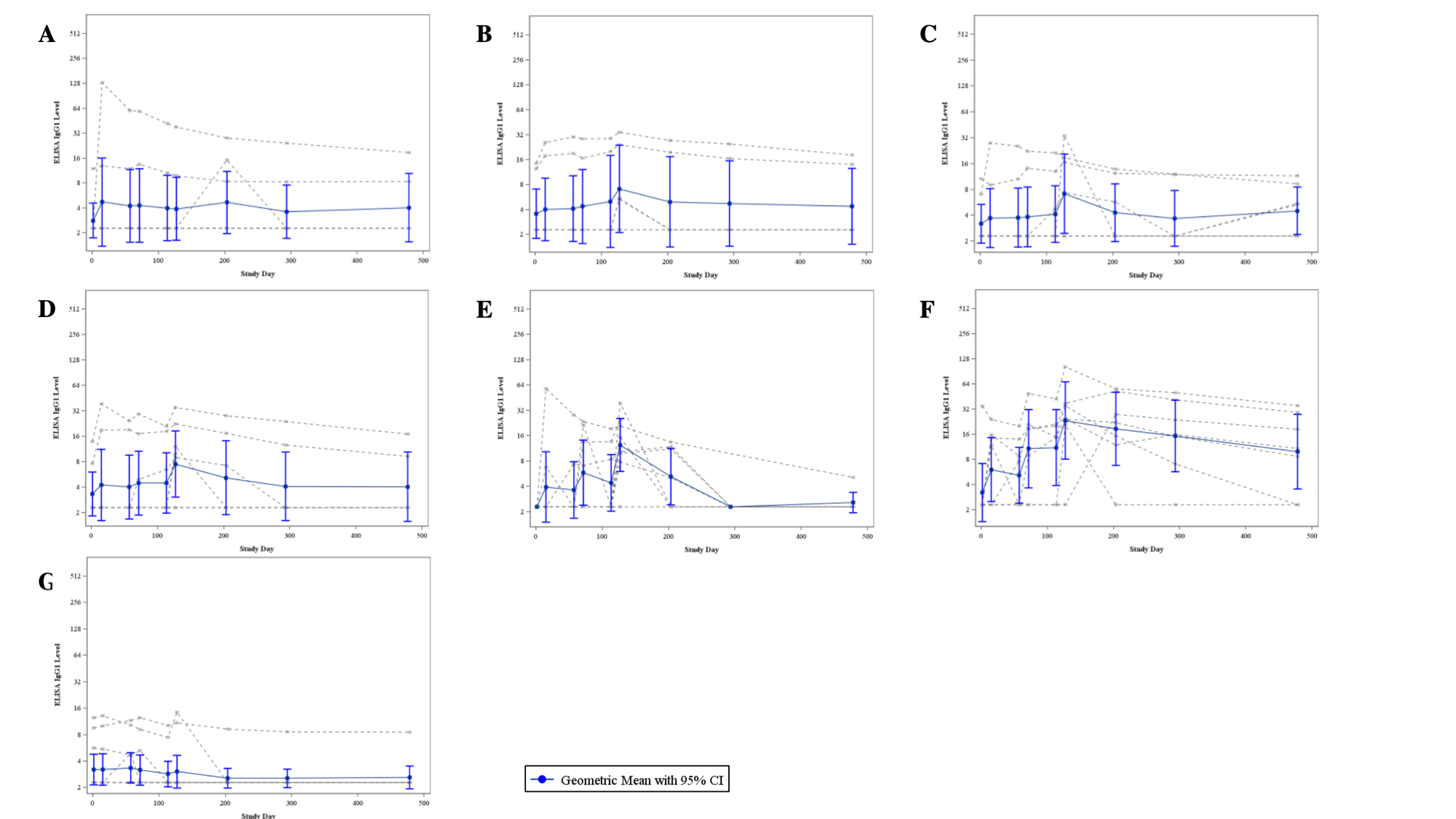

Supplement: S3 Fig — Individual study participant anti-Sm-TSP-2 ELISA IgG1 values over time: (A) 10 μg Sm-TSP-2/Alhydrogel; (B) 10 μg Sm-TSP-2/Alhydrogel with AP 10–701; (C) 30 μg Sm-TSP-2/Alhydrogel; (D) 30 μg Sm-TSP-2/Alhydrogel with AP 10–701; (E) 100 μg Sm-TSP-2/Alhydrogel; (F) 100 μg Sm-TSP-2/Alhydrogel with AP 10–701; (G) Euvax Hepatitis B vaccine. (TIF) [file pntd.0011236.s004.tif]

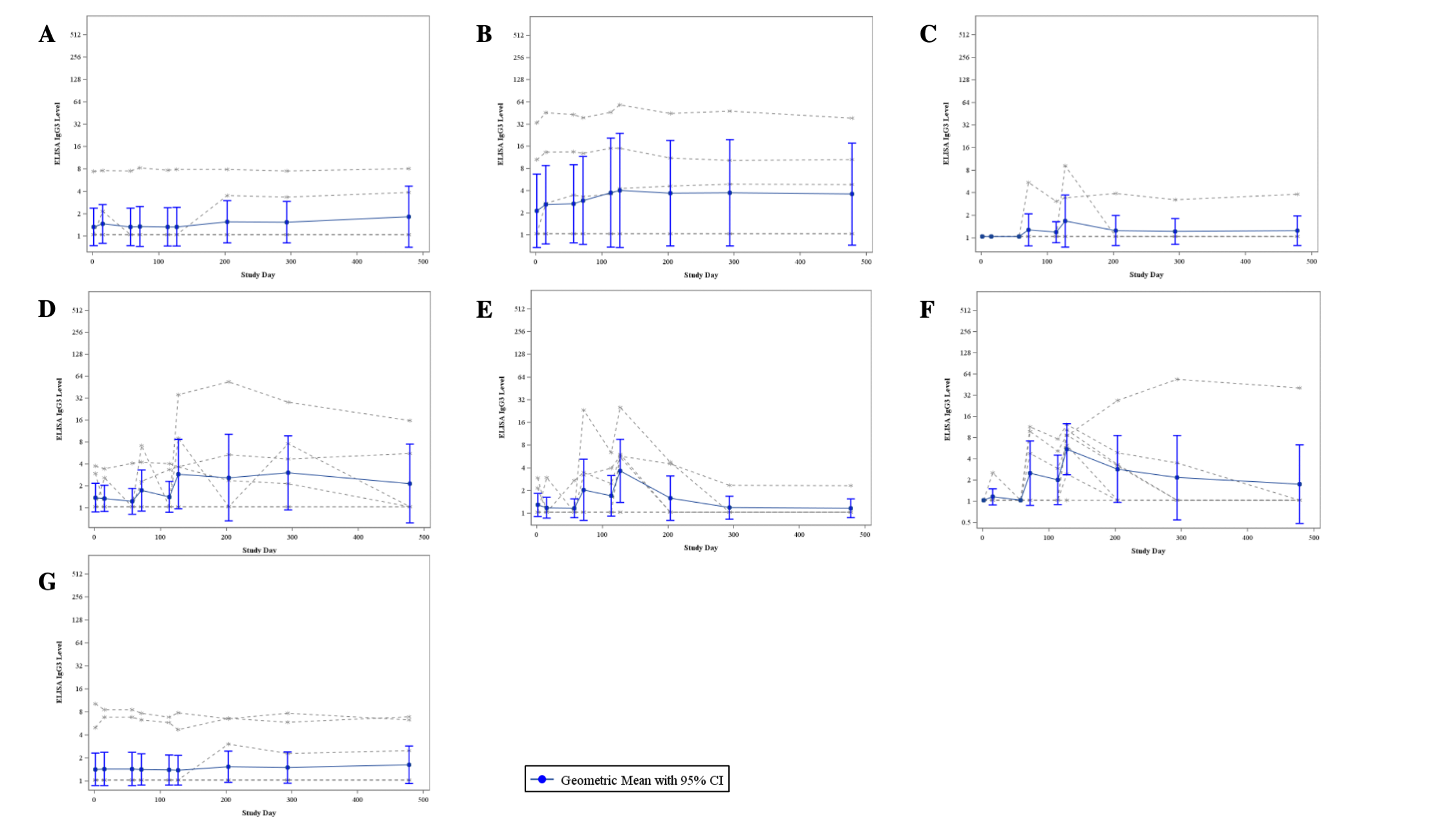

Supplement: S4 Fig — Individual study participant anti-Sm-TSP-2 ELISA IgG3 values over time: (A) 10 μg Sm-TSP-2/Alhydrogel; (B) 10 μg Sm-TSP-2/Alhydrogel with AP 10–701; (C) 30 μg Sm-TSP-2/Alhydrogel; (D) 30 μg Sm-TSP-2/Alhydrogel with AP 10–701; (E) 100 μg Sm-TSP-2/Alhydrogel; (F) 100 μg Sm-TSP-2/Alhydrogel with AP 10–701; (G) Euvax Hepatitis B vaccine. (TIF) [file pntd.0011236.s005.tif]

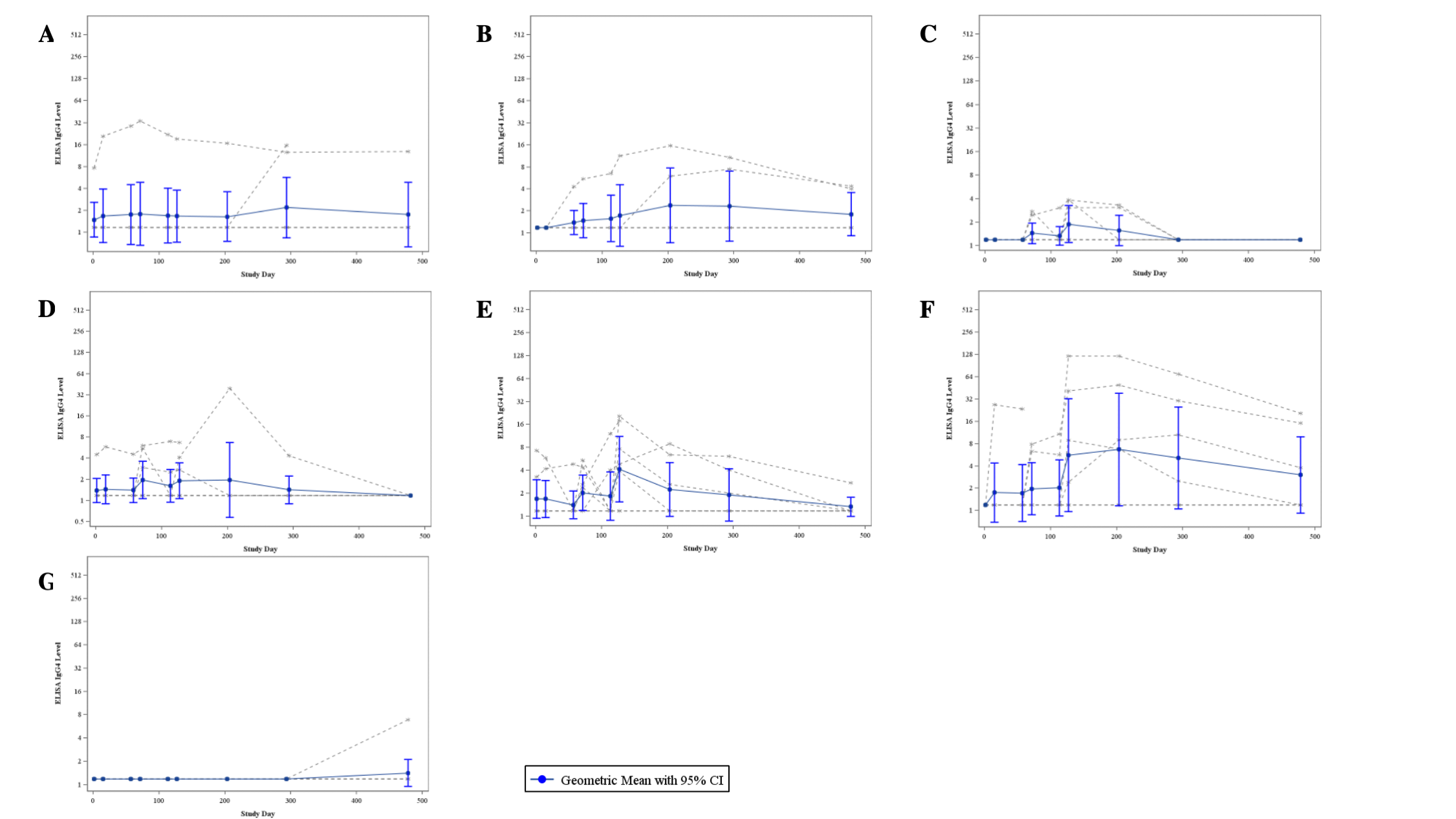

Supplement: S5 Fig — Individual study participant anti-Sm-TSP-2 ELISA IgG4 values over time: (A) 10 μg Sm-TSP-2/Alhydrogel; (B) 10 μg Sm-TSP-2/Alhydrogel with AP 10–701; (C) 30 μg Sm-TSP-2/Alhydrogel; (D) 30 μg Sm-TSP-2/Alhydrogel with AP 10–701; (E) 100 μg Sm-TSP-2/Alhydrogel; (F) 100 μg Sm-TSP-2/Alhydrogel with AP 10–701; (G) Euvax Hepatitis B vaccine. (TIF) [file pntd.0011236.s006.tif]
