# Supplementary material for: A randomized, controlled Phase 1b trial of the Sm-TSP-2 Vaccine for intestinal schistosomiasis in healthy Brazilian adults living in an endemic area
Source: PLoS Negl Trop Dis. 2023 Mar 30;17(3):e0011236. doi: 10.1371/journal.pntd.0011236 (PMC10089325; doi:10.1371/journal.pntd.0011236)
Supplement: S1 Table — (DOCX) [file pntd.0011236.s007.docx]

**S1 Table.** *S. mansoni* egg counts per gram of feces for participants who tested positive at baseline or study day 293, as measured by Kato Katz fecal thick smear.

| **Participant ID** | **Vaccine Group** | **Kato Katz Fecal Egg Count (epg)** | |
| --- | --- | --- | --- |
|  |  | Screening | Study Day 293 |
| CPA.04154 | 10 µg *Sm*-TSP-2/Alhydrogel | 12.0 | 0 |
| CPA.04155 | 10 µg *Sm*-TSP-2/Alhydrogel + AP 10-701 | 0 | 12.0 |
| CPA.04331 | 30 µg *Sm*-TSP-2/Alhydrogel + AP 10-701 | 12.0 | 0 |
| CPA.04701 | 100 µg *Sm*-TSP-2/Alhydrogel | XX | 0 |
| CPA.04704 | 100 µg *Sm*-TSP-2/Alhydrogel | XX | 0 |
| CPA.04694 | 100 µg *Sm*-TSP-2/Alhydrogel + AP 10-701 | 0 | 36.0 |
| CPA.04156 | Euvax-B | 18.0 | 0 |
