# Supplementary material for: A randomized, controlled Phase 1b trial of the Sm-TSP-2 Vaccine for intestinal schistosomiasis in healthy Brazilian adults living in an endemic area
Source: PLoS Negl Trop Dis. 2023 Mar 30;17(3):e0011236. doi: 10.1371/journal.pntd.0011236 (PMC10089325; doi:10.1371/journal.pntd.0011236)
Supplement: S2 Table — (DOCX) [file pntd.0011236.s008.docx]

**S2 Table.** **Summary statistics for the duration (days) of solicited adverse events, by vaccine group.**

|  | **Any Systemic Solicited AE** | | | | **Any Injection Site Solicited AE** | | | | **Any Solicited AE** | | | |
| --- | --- | --- | --- | --- | --- | --- | --- | --- | --- | --- | --- | --- |
| **Treatment Group** | **n** | **Mean** | **Median** | **Range** | **n** | **Mean** | **Median** | **Range** | **n** | **Mean** | **Median** | **Range** |
| Euvax B Vaccine (N=12) | 4 | 2.0 | 2.0 | 1, 3 | 3 | 7.0 | 5.0 | 2, 14 | 4 | 6.0 | 4.0 | 2, 14 |
| 10μg *Sm*-TSP-2/Alhydrogel (N=8) | 3 | 3.0 | 2.0 | 2, 5 | 5 | 4.8 | 1.0 | 1, 17 | 6 | 5.3 | 2.5 | 1, 17 |
| 10μg *Sm*-TSP-2/Alhydrogel with AP 10-701 (N=8) | 2 | 6.0 | 6.0 | 2, 10 | 5 | 1.8 | 2.0 | 1, 3 | 6 | 3.2 | 2.0 | 1, 10 |
| 30μg (N=8) *Sm*-TSP-2/Alhydrogel | 5 | 2.8 | 3.0 | 1, 4 | 6 | 2.2 | 2.0 | 1, 3 | 7 | 3.7 | 4.0 | 1, 6 |
| 30μg *Sm*-TSP-2/Alhydrogel with AP 10-701 (N=8) | 4 | 3.5 | 4.0 | 1, 5 | 8 | 5.0 | 5.0 | 1, 8 | 8 | 6.3 | 6.5 | 1, 10 |
| 100μg *Sm*-TSP-2/Alhydrogel (N=8) | 3 | 4.0 | 2.0 | 2, 8 | 6 | 4.5 | 5.0 | 2, 7 | 6 | 6.3 | 5.5 | 4, 12 |
| 100μg *Sm*-TSP-2/Alhydrogel with AP 10-701 (N=8) | 4 | 2.5 | 2.0 | 2, 4 | 8 | 6.8 | 6.5 | 3, 12 | 8 | 7.6 | 7.0 | 3, 12 |
| All Active^a^ (N=48) | 21 | 3.4 | 2.0 | 1, 10 | 38 | 4.4 | 3.5 | 1, 17 | 41 | 5.5 | 5.0 | 1, 17 |
| Note: AE = Adverse event; N = Number of participants in the Safety Population who received at least 1 dose of study product; n = Number of participants that reported at least one solicited AE.  ^a^ A pooled group of participants who received any dose of *Sm*-TSP-2/Alhydrogel with or without 5μg AP 10-701. | | | | | | | | | | | | |
