# Supplementary material for: A randomized, controlled Phase 1b trial of the Sm-TSP-2 Vaccine for intestinal schistosomiasis in healthy Brazilian adults living in an endemic area
Source: PLoS Negl Trop Dis. 2023 Mar 30;17(3):e0011236. doi: 10.1371/journal.pntd.0011236 (PMC10089325; doi:10.1371/journal.pntd.0011236)
Supplement: S3 Table — (DOCX) [file pntd.0011236.s009.docx]

**S3 Table.** **Listing of clinical laboratory adverse events experienced by study participants.**

| **Participant ID** | **Vaccine Group** | **Sex** | **Age (years)** | **Study Day** | **Parameter (unit)** | **Value** | **Related/**  **Unrelated** |
| --- | --- | --- | --- | --- | --- | --- | --- |
| CPA.04113 | 10 µg *Sm*-TSP-2/Alhydrogel | M | 46 | 57 | ALT (U/L) | 57 | Unrelated |
| CPA.04154 | 10 µg *Sm*-TSP-2/Alhydrogel | F | 22 | 113 | WBC (10^6^/L) | 11,030 | Unrelated |
| CPA.04107 | 10 µg *Sm*-TSP-2/Alhydrogel + AP 10-701 | M | 47 | 64 | ALT (U/L) | 56 | Unrelated |
| CPA.04317 | 30 µg *Sm*-TSP-2/Alhydrogel + AP 10-701 | M | 19 | 120 | WBC (10^6^/L) | 11,910 | Related |
| CPA.04324 | 30 µg *Sm*-TSP-2/Alhydrogel + AP 10-701 | F | 26 | 57 | Platelets (10^6^/L) | 458 | Unrelated |
| CPA.04324 | 30 µg *Sm*-TSP-2/Alhydrogel + AP 10-701 | F | 26 | 57 | Platelets (10^6^/L) | 479 | Unrelated |
| CPA.04697 | 100 µg *Sm*-TSP-2/Alhydrogel | M | 22 | 113 | WBC (10^6^/L) | 16,380 | Unrelated |
| CPA.04725 | 100 µg *Sm*-TSP-2/Alhydrogel + AP 10-701 | F | 21 | 8 | ALT (U/L) | 140 | Unrelated |
| CPA.04728 | 100 µg *Sm*-TSP-2/Alhydrogel + AP 10-701 | M | 36 | 57 | Creatinine (mg/dL) | 1.66 | Unrelated |
| CPA.04732 | Euvax-B | F | 25 | 8 | WBC (10^6^/L) | 12,680 | Unrelated |
| CPA.04732 | Euvax-B | F | 25 | 57 | WBC (10^6^/L) | 11,950 | Unrelated |
| CPA.04732 | Euvax-B | F | 25 | 57 | Platelets (10^6^/L) | 484 | Unrelated |
| CPA.04312 | Euvax-B | M | 45 | 64 | WBC (10^6^/L) | 12,040 | Unrelated |

*Note: ALT = alanine aminotransferase; WBC = white blood cell count*
