# Supplementary material for: A randomized, controlled Phase 1b trial of the Sm-TSP-2 Vaccine for intestinal schistosomiasis in healthy Brazilian adults living in an endemic area
Source: PLoS Negl Trop Dis. 2023 Mar 30;17(3):e0011236. doi: 10.1371/journal.pntd.0011236 (PMC10089325; doi:10.1371/journal.pntd.0011236)
Supplement: Attachment — Submitted filename: Response to Reviewers_R1.pdf [file pntd.0011236.s010.pdf]

THE GEORGE WASHINGTON UNIVERSITY
WASHINGTON, DC

Department of Medicine

Responses to Reviewer Comments:

Reviewer #1: **This is a very well written manuscript that describes the successful completion of Phase 1b trial of the *Sm*-TSP-2 vaccine against schistosomiasis. First, I will like to congratulate the authors for validating the safety of the vaccines using a randomized, observer-blind, controlled phase 1b clinical trial in 60 healthy adults living in a region of Brazil with ongoing *S. mansoni* transmission, as well as showing that the two adjuvanted *Sm*-TSP-2 vaccines (+alum or +alum/AP 10-701) are minimally reactogenic, and importantly both elicited significant IgG and IgG subclass responses against the vaccine antigen after the third dose (in particular when 100 ug of the antigen was used). The outcomes of this study have led to the initiation of a Phase 2 clinical trial of this vaccine in an endemic region of Uganda.**

Authors' Response: Thank you for the kind assessment.

I have few minor recommendations that might enhance the value of this study.

1. **Although the authors have clearly described the experimental details of the clinical trial and its outcomes (safety and immunogenicity), I recommend that the authors share some of their immunogenicity data more plainly, which may help other researchers who are considering conducting clinical trials to evaluate their own vaccine studies. Specifically, and for example, how does 4-fold increase in arbitrary units (AU) (using 1:4000 dilution of sera) translates to actual ELISA ODs; seroresponse was defined as >4-fold rise over baseline.**

   If I understand it correctly, analysis of all the Standard Reference Serum (SRS) control testing (Figure S1) implies that a 10-100 AU range is associated with OD range of 0.5-3.5, while 1:4000 dilution of the SRS is about an OD of 0.5. As the antigenicity outcomes were presented only as AU (Figs 4 and 6) and fold change (Fig. 5) it is very hard to recognize the actual levels (OD at 1:4000) of the IgG and/or IgG1 and IgG3 responses that were elicited, which have made the 100ug adjuvanted vaccines more potent than the 10 or 30 ug doses (Figure 4; Table 2); what are for example the ODs of 16.2 fold AU (day 293) vs AU of 25 (day 127) of the *Sm*-TSP-2 + alum/AP 10-701 vaccine vs. the other groups and vs. the baseline of 4-fold AU? Adding such pertinent supporting information where appropriate could enhance the value of the comparative immunogenicity studies across the 6 experimental vaccine groups.

   I also wonder whether presenting the data in the various groups separately (like in figure S2) instead a congregated format of the 7 groups (Figure 4 and Figure 6) might be more informative and show more clearly the kinetics in each group's participants and their associated error bars. As presented, there is too much indistinguishable data in each graph.

   Authors' Response:

   A deliberate decision was made to present the IgG and IgG subclass results using Arbitrary Units instead of OD values, specifically to permit comparison of immunogenicity results between the 6 experimental vaccine groups, as well as between clinical trials. Use of a Standard Reference Serum (SRS) allows for standardization across different ELISA runs done on different days and/or by different

operators, as well as using different spectrophotometers. As described in the manuscript, each ELISA plate included serial dilutions of the SRS that permitted a standard calibration curve to be fitted and from which test sera OD readings could be interpolated to derive AUs values. This methodology allows for much more robust standardization, repeatability, and comparability between ELISA plates, runs, operators, and instruments than using OD readings. In lieu of using mass values (e.g., μg/ml) of antibody, which requires extensive assay validation, reporting ELISA binding antibody results in arbitrary units, derived from a SRS, is standard for clinical trials of investigational vaccines [1-3].

Regarding Figures 4 and 6, we believe that including all vaccine groups on the same graph allows for a clearer comparison between the immune responses of the different vaccine groups. As suggested, the supplemental S2 Fig does display the individual IgG responses by vaccine group (as well as the geometric mean and error bars) in separate panels. Additional figures showing the individual IgG subclass responses by vaccine group have been added as supplemental material, as requested (S3 Fig, S4 Fig, and S5 Fig for IgG1, IgG3, and IgG4 responses).

2. **Second, the vaccine antigen was selected "based on its unique recognition by cytophilic antibodies in putatively immune individuals living in areas of ongoing *S. mansoni* transmission in Brazil, and preclinical studies in which vaccination with *Sm*-TSP-2 protected mice following infection".**

   I wonder if a functional assay was developed that can correlate directly and specifically the increased immunogenicity with the functionality of the anti-*Sm*-TSP-2 IgG and cytophilic antibodies elicited by the best vaccine formulation. If there is such an assay, was it done, and if not, a discussion pointing to such an experimental gap should be considered. In reference 14, it was shown that the putatively immune individuals have significant elevated mean IgG1 and IgG3 responses (1:100, ELISA) against TSP-2 of OD ~1.5 and 1, respectively, in comparison to the chronically infected individuals. What are the optimal titers of induced TSP-2 cytophilic antibodies that can be associated indirectly with protection?

   Stating simply in the discussion that the vaccine elicited IgG responses "consisting primarily of IgG1, which parallels the unique and presumably protective humoral immune response to *Sm*-TSP-2 observed in putatively resistant individuals resident in the same *S. mansoni* endemic area of Brazil" is insufficient as it is not based on direct functional evidence and therefore might be somewhat misleading that the desired protective humoral responses having a critical role in ADCC were actually elicited.

   Authors' Response:

   A functional assay has not yet been developed to correlate immunogenicity with functionality of anti-*Sm*-TSP-2 IgG and cytophilic antibodies. We have therefore revised the sentence in question to state that the vaccine elicited IgG responses "consisting primarily of IgG1, which mirrors the humoral immune response to *Sm*-TSP-2 observed in putatively resistant individuals resident in the same *S. mansoni* endemic area of Brazil," to avoid any suggestion that we are claiming that a protective immune response was induced in study volunteers. Such protection can only be definitely evaluated

in Phase 3 efficacy clinical trials. A statement has also been added to the final paragraph of the Discussion to specifically state that the functionality of the induced antibodies was not assessed.

3. **Thirdly, it is not clear which formulation and dose(s) was selected for the Phase 2 clinical trial; Sm-TSP-2/Alhydrogel or the *Sm*-TSP-2/Alhydrogel + AP 10-701, and what readout are being expected. Such statements might be instructive.**

   Authors' Response:

   The dose and formulation that is being tested in the Phase 2 trial currently underway in Uganda is 100 µg *Sm*-TSP-2/Alhydrogel plus 5 µg AP 10-701. However, the decision on this dose and formulation was not only based on the results of the clinical trial reported herein, but also based on data from a dose-escalation Phase 1 trial conducted in the same Ugandan population (not yet published). As suggested, we have added a paragraph to the Discussion to state the dose and formulation being tested in the Phase 2 trial, as well as the primary efficacy endpoint (rate or re-infection with *S. mansoni*). In the ongoing Phase 2 trial, adult volunteers who have tested positive for *S. mansoni* by fecal microscopy are treated with praziquantel before being vaccinated with the *Sm*-TSP-2 vaccine or an active comparator. The primary endpoint will be the rate of re-infection over an 18-month period of follow-up.

Reviewer #2: **Diemert and co-authors report a well conducted Phase Ib study of *Sm*-TSP-2. The following are some considerations for improving the manuscript:**

1. **Introduction:**
   Para 3 from line 103. The key data describing the recognition of *Sm*-TSP by putatively immune humans is not referenced (line 117). Reference 14 does not match the mouse challenge model.

   Authors' Response:

   The reference to the manuscript describing the recognition of *Sm*-TSP-2 by putatively immune humans has been added as requested. The reference to the mouse challenge model has also been corrected.

2. **Methods.**
   It appears that Ag-specific IgE was only tested for at enrolment. I would be curious if Ag-specific IgE was generated by the vaccine.

   Authors' Response:

   Antigen-specific IgE was only tested for during screening since the presence of such antibodies was exclusionary due to the possible risk of inducing immediate-type allergic reactions. Study participants were not tested for anti-*Sm*-TSP-2 IgE after vaccination since recombinant protein vaccines do not usually induce significant levels of this antibody isotype, and IgE antibodies are not expected to be

important in protection against infection. Lastly, allergic reactions were not observed after the second or third vaccinations, which may have been seen had significant levels of antigen-specific IgE been induced by previous vaccine administrations.

3. **Results**
Baseline parasitolgical investigation. As the authors highlight, the TSP experience with pre-sensitization has been an issue of interest. A more comprehensive reporting of the baseline parasitologic findings would be appropriate (beyond kato katz for Sm and Ag-specific IgE). A more comprehensive parasitologic evaluation is described in the methods. Was baseline schisto serology done on study subjects? Any baseline parasitologic data should be presented (even if in supplement).

Authors' Response:

The only parasitological investigations performed at baseline were microscopic fecal examinations (Kato Katz fecal thick smear) and anti-*Sm*-TSP-2 serology (IgG, IgG subclasses, and IgE). Only screened volunteers with undetectable anti-*Sm*-TSP-2 IgE were enrolled, so baseline values for all study participants were by definition undetectable. Baseline IgG and IgG subclass levels are shown in Table 2 and Figures 4 and 6. Antibody responses to crude *S. mansoni* extracts were not performed at any point during the study (neither at screening nor during the trial). The fecal egg counts (by Kato Katz) for the five participants who were positive for *S. mansoni* at baseline and the two who tested positive at the end of the follow-up period, have been added as supporting information (S1 Table). Pre-sensitization to potential helminth vaccine antigens is due to induction of antigen-specific IgE antibodies due to prior infection. For example, this was observed in a Phase 1 trial of the recombinant *Na*-ASP-2 hookworm vaccine, in which some volunteers in an endemic area of Brazil who were treated for infection with *Necator america*nus and then vaccinated with *Na*-ASP-2 developed urticaria due to baseline levels of anti-*Na*-ASP-2 IgE antibodies [4]. However, in the Phase 1 trial of *Sm*-TSP-2 reported herein, not only were volunteers excluded if they had detectable IgE to *Sm*-TSP-2 during screening, no participant developed urticaria or other immediate-type hypersensitivity reactions due to vaccination during the study.

4. **Line 392 "renal dilatation" is not something I don't recognize. Suggest omit and just state "rigid ureterorenal lithotripsy".**

Authors' Response:

This has been changed as suggested.

5. **Clinical Lab AEs (from line 457): Actual lab values should be given in text (or in supplement)**

Authors' Response:

These data have been added as supporting information (Table S3), as requested.

6. **Table 2:**
   This does not display the key data well. Graphical presentation of key findings would be better. I recognize that 63 scatterplots would be excessive (!) but 7 serial scatterplots of actual Ab level would show the data better. Raw data should be presented in supplement, or a data sharing statement included.

   Authors' Response:

   Raw antibody data have been uploaded to the Dryad Digital Repository. This is now described in the manuscript's Data Availability Statement. The data presented in Table 2 are presented graphically in Figure 4. In addition, individual scatterplots of IgG responses have been added as supporting information (Fig S3).

7. **Discussion**
   Do the authors predict cross-reactive responses to Sh-TSP?

   Authors' Response:

   The possibility of *Sm*-TSP-2 inducing cross-reactive antibody or other immune responses to *S. haematobium* TSP-2 is entirely theoretical at this point, as no empiric studies have been conducted. However, as reported by Mekonnen et al, the amino acid sequence similarities between the *S. haematobium* tetraspanins, including Sh-TSP-2, and their *S. mansoni* homologs ranges from 71–93% when entire open reading frames are compared and 70.2–84% when only the large extracellular loop regions are compared [5]. Therefore, it is likely that IgG antibodies induced by the *Sm*-TSP-2/Alhydrogel vaccine will cross-react with *Sh*-TSP-2. This will be tested in the future. A paragraph has been added to the Discussion regarding the as-yet-untested possibility that the antibody response to *Sm*-TSP-2 may be cross-reactive with *Sh*-TSP-2.

8. **Do the authors have any data from animal experiments or seroepidemiologic data to infer what a likely "protective" Ab titer would be?**

   Authors' Response:

   As the COVID-19 pandemic has demonstrated, establishing an antibody level threshold that is protective can be extremely difficult, even after the huge Phase 3 trials that were conducted for SARS-CoV-2 vaccines. Although seroepidemiologic data has demonstrated an association between IgG antibodies to *Sm*-TSP-2 and protection from infection in putatively immune individuals in Brazil who are repeatedly exposed to the parasite but remained uninfected [6], this is just an association in that putatively resistant individuals had higher levels of antibody to *Sm*-TSP-2 compared to those who were chronically infected. However, in this observational study, anti-*Sm*-TSP-2 IgG antibodies may have been only one of several protective mechanisms. Furthermore, establishing a correlate of protection will only be possible in Phase 2 and 3 clinical trials of the vaccine, in which post-vaccination IgG levels are correlated to subsequent protection from infection during the follow-up period. We did not make any changes to the manuscript relative to this point.

9. **The authors imply that one of the formulations is going into Phase II. Which is it and what is the rationale for this decision?**

   <u>*Authors' Response*</u>:

   The dose and formulation that is being tested in the ongoing Phase 2 trial in Uganda is 100 µg *Sm*-TSP-2/Alhydrogel plus AP 10-701. However, the decision on this dose and formulation not only based on the results of the clinical trial reported herein, but also based on data from a dose-escalation Phase 1 trial conducted in the same Ugandan population. Nevertheless, a paragraph has been added to the Discussion to state the dose and formulation being tested in the Phase 2 trial, as well as the primary efficacy endpoint (rate or re-infection with *S. mansoni*). In the ongoing Phase 2 trial, adult volunteers who have tested positive for *S. mansoni* by fecal microscopy are treated with praziquantel before being vaccinated with the *Sm*-TSP-2 vaccine or an active comparator. The primary endpoint will be the rate of re-infection over an 18-month period of follow-up.

References cited:

1. Jariwala AR, Oliveira LM, Diemert DJ, Keegan B, Plieskatt JL, Periago MV, et al. Potency testing for the experimental Na-GST-1 hookworm vaccine. Expert Rev Vaccines. 2010;9(10):1219-30. Epub 2010/10/07. doi: 10.1586/erv.10.107. PubMed PMID: 20923271.
2. Giersing BK, Dubovsky F, Saul A, Denamur F, Minor P, Meade B. Potency assay design for adjuvanted recombinant proteins as malaria vaccines. Vaccine. 2006;24(20):4264-70. PubMed PMID: 16767804.
3. Brelsford JB, Plieskatt JL, Yakovleva A, Jariwala A, Keegan BP, Peng J, et al. Advances in neglected tropical disease vaccines: Developing relative potency and functional assays for the Na-GST-1/Alhydrogel hookworm vaccine. PLoS Negl Trop Dis. 2017;11(2):e0005385. Epub 2017/02/13. doi: 10.1371/journal.pntd.0005385. PubMed PMID: 28192438; PubMed Central PMCID: PMCPMC5325600.
4. Diemert DJ, Pinto AG, Freire J, Jariwala A, Santiago H, Hamilton RG, et al. Generalized urticaria induced by the Na-ASP-2 hookworm vaccine: implications for the development of vaccines against helminths. J Allergy Clin Immunol. 2012;130(1):169-76 e6. Epub 2012/05/29. doi: 10.1016/j.jaci.2012.04.027. PubMed PMID: 22633322.
5. Mekonnen GG, Tedla BA, Pearson MS, Becker L, Field M, Amoah AS, et al. Characterisation of tetraspanins from Schistosoma haematobium and evaluation of their potential as novel diagnostic markers. PLoS Negl Trop Dis. 2022;16(1):e0010151. Epub 20220124. doi: 10.1371/journal.pntd.0010151. PubMed PMID: 35073344; PubMed Central PMCID: PMCPMC8812969.
6. Tran MH, Pearson MS, Bethony JM, Smyth DJ, Jones MK, Duke M, et al. Tetraspanins on the surface of Schistosoma mansoni are protective antigens against schistosomiasis. Nat Med. 2006;12(7):835-40. PubMed PMID: 16783371.
